# Notes and News

**Published:** 1891-11-14

**Authors:** 


### NOTES AND NEWS.

COLLECTED AND COMMUNICATED.

THE second volume of testimony before the Lords' Committee of the Metropolitan Hospitals is now out. It is still larger than the first report, running to over 800 pages. If the present systems are really unsatisfactory, it is to be hoped that this mass of material will not daunt those who really have some definite action in view. If the whole matter is to end in smoke, the weighty volumes will be sad memorials of wasted time. During some of the last sittings of the Committee the noble lords present looked as if they had heard quite enough on the subject; indeed, their weary faces provoked our pity.

HIS Royal Highness the Prince of Wales, who is President of St. Bartholomew's Hospital, took the chair at a meeting held in the Great Hall of that institution, for the purpose of receiving a portrait of Sir Sydney Waterlow, who has been its Treasurer for 18 years. The portrait, which is full length, was painted by Professor Herkomer, R.A., and is marked by all the vigour and truthfulness which characterise the portraits of that distinguished artist. It is to be placed immediately over the entrance to the Great Hall. On the frame of the picture is the following inscription:—"1891.—To Sir Sidney Hedley Waterlow, Bart., Treasurer of St. Bartholomew's Hospital, 1874, to——. Presented to the Hospital by Subscription of His Royal Highness, President, the Governors, and the Members of the General Staff." His Royal Highness, in the course of his speech mentioned the progress made by the institution, and said that since 1873 the increase of income from endowments had risen from £42,000 net to £65,000 net in 1891. There has been a reorganisation of the nursing arrangements, a systematic instruction of the nursing staff, and a large increase in their numbers. In 1873 the female staff was 116, it is now 250, and a body of nurses has been trained for supplying nursing to the public. The medical staff has also increased from 18 in 1873 to 39 last year. The medical school buildings have been almost entirely re-erected at a cost of £50,000. There has also been an increasing number of entrances of new students. In the five years before 1873 the average number was 86; in the five years last ended it has been 120. We had first a convalescent home for 34 males. It was at Highgate, in a house given by Sir Sydney Waterlow and adapted to the purpose at his own cost. (Cheers.) We have now a convalescent home at Swanley capable of holding 70 patients—45 males and 25 females. It was built by Mr. Kettlewell at a cost of £15,000, and was opened by the Prince in 1885.

YEAST has been successfully tried as a remedy for typhoid fever by Dr. Embling, Dr. Lemprière, and Dr. Thomson, of the Alfred Hospital, Melbourne. Thirty-seven cases were treated, ten being severe, the temperatures reaching 104 deg.; eight were moderate, the temperatures being 103 deg.; eleven were mild, and eight were very mild, the temperatures reaching 102 deg. In every case the recovery took place without a relapse. There is a theory to the effect that relapses are due to reinfection from the intestine, and Dr. Thomson remarks in his report that yeast should destroy the bacilli in the intestinal tube, and so prevent reinfection.

THE correspondence between the Memorialists and the authorities of the Manchester Infirmary still continues. The Trustees are most emphatic in their desire that the Board shall realise that they will encounter strenuous opposition should they endeavour to carry out the proposed extension of the institution on the present site. They have no less good authority on their side than Florence Nightingale, who, in "Notes on Hospitals" and other works, says: "If the recovery of the sick is to be the object of hospitals, they will not be built in towns. Town sites should be avoided as far as possible. If the crowding of healthy men has its dangers, the bringing together within a confined area of many sick persons is far more perilous. As soon as a patient can get out of his ward he should do so; therefore, open verandahs, sheltered gardens, are most important."

DR. BUDIN, the well-known obstetric doctor at the Charité Hospital in Paris, has now under his charge a new maternity department at that institution, which it is prophesied will not only greatly benefit those resorting to it as patients, but also a large class who hitherto have not been able to obtain such advantages in instruction. The old department occupied an unfavourable position on the second storey of a block containing medical and surgical wards on either side—notwithstanding this the results were remarkably satisfactory. An unfavourable position was not the only objection to the department. It was found to be far too small, and the Assistance Publique were at the expense of boarding out many patients with midwives, at a cost of more than double the expenditure per head at the hospital. It was finally decided to build a new department, and this is now complete in all respects, and is admirably arranged. There is a service of 55 beds, and all necessary arrangements for a complete course of instruction, both theoretical and practical. Dr. Budin will devote himself mainly to teaching, and hopes that many of the students who now migrate to Vienna for practical work, will find all they want nearer home.

A WELL-KNOWN Birmingham physician, Sir James Sawyer, in an address before the Midland Counties Chemists' Association, drew attention to the mistaken tendency of the public, to demand cheap dispensing. "The patient," he observes, "does not mind paying a big fee to the physician for his prescription, but he often grudges a small fee to the pharmacist." Now, is this the case? It is, we should say, not a small but a big fee that is grudged to the pharmacist. People have discovered that they can procure at the stores drugs for which the chemists charge them twice or thrice as much. Rightly or wrongly, the public has got it into its head that the profits made by chemists are out of all proportion. The doctor will prescribe, and pharmacists will "make up" concoctions for which the latter will blandly ask considerably more than is asked at the humble drug store. The *Globe* remarks that there are fashionable chemists whose profits are probably at the rate of 100 per cent., if not of more. Moreover, there are doctors and physicians who play into the hands of the chemists whom they patronise. There are, of course, honest pharmacists; but, speaking generally, chemists' prices are, apparently, purely arbitrary.

